# Validity and Reproducibility of an Electronic Food Frequency Questionnaire in Argentinian Adults

**DOI:** 10.3390/nu16111564

**Published:** 2024-05-22

**Authors:** Rocio Victoria Gili, Sara Leeson, Belén Carlino, Ismael Alejandro Contreras-Guillén, Daniel Xutuc, Marcia Cristina Teixeira Martins, María del Pilar Díaz, Gina Segovia-Siapco, Sandaly Oliveira da Silva Pacheco, Fabio Juliano Pacheco

**Affiliations:** 1 Interdisciplinary Center for Research in Health and Behavioral Sciences, School of Medicine and Health Sciences, Universidad Adventista del Plata, Libertador San Martín 3103, Argentina; sara.leeson@uap.edu.ar (S.L.); belen.carlino@uap.edu.ar (B.C.); ismael.contreras@uap.edu.ar (I.A.C.-G.); daniel.xutuc@uap.edu.ar (D.X.); 2 School of Nutrition, Faculty of Medical Sciences, Universidad Nacional de Córdoba, Córdoba 5016, Argentina; pdiaz@fcm.unc.edu.ar; 3 School of Agricultural Sciences, Food Sciences and Engineering, Universidad Nacional de Entre Ríos, Oro Verde 3100, Argentina; 4 Graduate Department, Universidad Adventista de Chile, Camino a Las Mariposas, Chillán 11771, Chile; marciactm@yahoo.com.br; 5 Institute of Health Sciences Research (INICSA), School of Medical Sciences, Universidad Nacional de Córdoba, Córdoba 5016, Argentina; 6 School of Public Health, Loma Linda University, 24951 Circle Dr. Nichol Hall, Loma Linda, CA 92350-1718, USA

**Keywords:** electronic food frequency questionnaire, validity, reproducibility, 24 h dietary recalls, dietary assessment, Argentina

## Abstract

This study aimed to validate a semiquantitative electronic food frequency questionnaire (eFFQ) in estimating the intake of a comprehensive list of nutrients and bioactive compounds among adults from six regions of Argentina using multiple 24 h dietary recall (24HR) as a reference. A total of 163 adults completed two administrations of the eFFQ and four 24HRs. The paired *t*-test/Wilcoxon signed-rank test, Spearman/Pearson correlations, cross-classification, weighted kappa statistics, and Bland–Altman plots were employed to determine relative validity. To determine reproducibility, intraclass correlations (ICC), cross-classification, and weighted kappa statistics were calculated. For relative validity, crude correlations ranged from 0.15 to 0.57; energy adjustment and de-attenuation slightly improved most of these correlations. In cross-classification analysis, agreements within one quintile adjacent to exact agreement (EA ± 1) ranged from 52.2% to ~74%; extreme misclassifications were < 7%. For reproducibility, the crude ICC ranged from 0.29 to 0.85, showing moderate to good correlations for most nutrients. Cross-classification analysis showed agreement levels for the EA ± 1 quintile of 70.6% to 87.7%. Weighted kappa values ranged from 0.21 to 0.62. The results show that this eFFQ is relatively valid in ranking adults according to their nutrient intake and has an acceptable reproducibility, yet it slightly overestimates the intake of most nutrients.

## 1. Introduction

Non-communicable diseases (NCDs), such as cardiovascular diseases, cancers, respiratory diseases, and diabetes, are currently associated with more than two-thirds of all deaths worldwide and 86% of premature deaths that occur in low- and middle-income countries [1]. One of the predominant causes of the growing burden of NCDs is an unhealthy diet as demonstrated by various epidemiological studies that explore these diseases and their relationship with dietary patterns [2,3] and other diet-associated factors [4,5,6]. In Latin America, recent research has examined the relationships between dietary habits and NCDs [7,8,9,10,11,12]. Argentina experiences important sociocultural and economic inequalities that overburden the healthcare system and this scenario demands further studies in dietary lifestyle habits to address the health-risk behaviors and to generate strategies to mitigate NCDs [13,14].

The food frequency questionnaire (FFQ) is a widely used instrument in epidemiological studies to evaluate the typical or usual diet of people and it is one of the most used approaches in nutritional research [15]. In larger studies, they are relatively simple and less expensive to administer than other methods such as the dietary history and the 24 h dietary recall (24HR) [16]. Some FFQs developed in Argentinian cities, such as for residents of Córdoba, Buenos Aires, and Rosario, have been tested for validity and reproducibility [17,18,19]. However, none of these dietary assessment tools are electronic and national in scope. Because the FFQ may be prepared for a specific context, it is essential to document the validity and reproducibility of any new FFQ developed for specific population groups [18,20].

In recent years, the number of studies that collect data using online/electronic tools has increased. Technology has afforded the design of data collection tools including dietary assessment instruments in epidemiological studies. The use of digital instruments has several advantages including reduced time and cost spent on fieldwork, fewer potential interviewer biases, automatic collection and download of large data sets, easy data cleaning, real-time data coding, and automated intake calculations [21,22,23,24]. These advantages greatly optimize the work of researchers [25]. While traditional dietary assessment methods can be supplemented by food photo atlases to aid portion size recognition and estimation [26,27], online dietary assessment methods can also be designed to incorporate photographs of food and utensils for portion sizes. Carlsen et al. (2021) indicate that using images with portion sizes from digital media can facilitate the cognitive task of correctly choosing the portions consumed [28].

Online dietary assessment tools can be adapted to different geographic populations and can be accessed remotely, allowing better access and exploration compared to traditional methods [29]. The use of online dietary assessment methods can be more appreciated in Argentina where different regions of the country are geographically distant and a large proportion of the population are mobile-phone users [30].

We developed a semiquantitative electronic FFQ (eFFQ) designed for use in future epidemiological studies that are meant for tailor-made health interventions. This study aimed to validate the ability of this eFFQ to estimate the intake of nutrients and bioactive compounds among adults from the six main regions of Argentina using multiple 24HR as the standard. 

## 2. Materials and Methods

### 2.1. Study Population

Adults 21 years and older and residing for at least 3 years in the same region of Argentina were included in this study. Participant recruitment was carried out from the 6 main regions of Argentina: Northeast (the states of Misiones, Chaco, and Formosa); Northwest (the states of Salta and Tucumán); Midwest (the states of San Luis and Mendoza); Central (the states of Entre Ríos, Santa Fe, and Córdoba); the Buenos Aires region; and Southern (the states of Rio Negro, Neuquén, and Chubut). Potential participants were contacted via telephone or in person and invited to the study through Adventist institutions such as schools, churches, hospitals, and healthcare clinics.

Participants who did not fully complete the questionnaires, women with current or recent pregnancies, and those who had changed their usual diet for medical or other reasons during the past year were not included in the study. Also, participants who reported a total energy intake outside the range of 500–4000 kcal were excluded from the analysis [31]. The final sample of participants for the validation and reproducibility study was 163 subjects.

The study was evaluated and approved by the Research and Ethics Committee of the Adventist University of River Plate School of Medicine (resolution #1.7-8/2016), affiliated with the National Registry of Health Research (#237), Ministry of Health, Argentina. Participants were included in the study by invitation and acceptance of the terms of the informed consent. All procedures associated with this study were conducted following the international ethical standards proposed by the Helsinki protocol for human research. 

### 2.2. Study Design

**Figure 1:**
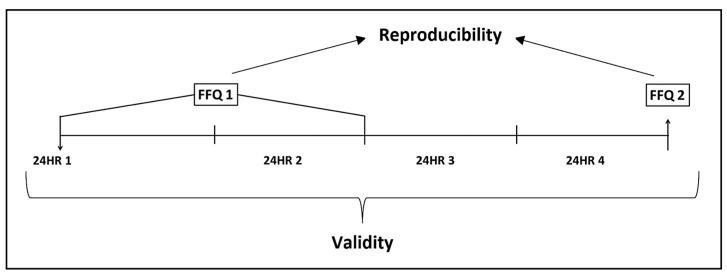
Diagram for data collection of food intake.

This study follows the guidelines and recommendations for reporting nutritional epidemiology and dietary assessment research described in the statement Strengthening the Reporting of Observational Studies in Epidemiology—Nutritional Epidemiology (STROBE-nut) [32]. The study was cross-sectional with multiple 24HRs and repeated administration of the developed eFFQ, and Figure 1 shows a diagram of the data collection process. Four 24HRs were collected for approximately a year, two during the spring to summer months, and two during the autumn to winter months. The eFFQ was self-administered at two time points which were about 12 months apart. The eFFQ was first administered at around the time the first and second 24HRs were collected and was administered again after participants completed the third and fourth 24HRs. Socio-demographic data (i.e., age, sex, occupation, education, and marital status), and self-reported anthropometric measurements (weight, height, and waist circumference) were also obtained. 

### 2.3. Reference Method: Multiple 24HR

The 24HR was used as a reference method to determine the validity of the eFFQ. The 24HR interviews were carried out when the dietary intake of the previous day reflected usual or habitual intake. If this was not the case, then the interview was postponed until later, considering that the data would reflect an atypical day in the consumption of food and beverages. The collection of 24HR was carried out by researchers, trained dietitians, and advanced dietitian students with the aid of the MAR24, an automated tool for 24HR previously developed by our group [33]. MAR24 uses the United States Department of Agriculture (USDA) 5-step multiple-pass method for dietary recall [34], consisting of a quick list of food items consumed; questions about commonly forgotten foods; detailed reports about the time and place where the food was consumed; food descriptions and amounts consumed estimated by a photo album of kitchen utensils; and a final review.

A total of four 24HRs were collected per person, two representing the food intake over the weekend—a Saturday and a Sunday—and two others on weekdays. For quality control, all the 24HR interviews were recorded using the Express Talk tool (NCH Software, Australia, Version 4.36). A random selection of 24HR audio records was regularly assessed by the investigators (S.L. or R.G.) to ensure data were properly collected and uploaded into the MAR24 database. The MAR24 tool was used to calculate the macronutrient and micronutrient composition of intakes reported in the 24HR as described elsewhere [33]. 

### 2.4. Development of Electronic Food Frequency Questionnaire 

A semiquantitative FFQ was developed in an electronic format and was designed to assess the habitual intake of foods that reflect regional diversity in Argentina. For the initial selection of the eFFQ food list, the basic structure of food items from a previously validated FFQ for Argentinian adults was considered [19]. Our group carried out a survey to further determine what foods to add to the food list. This survey consisted of 2 interviews with 60 participants from the 6 Argentinian regions, from whom a total of 120 interviewer-administered 24HRs were collected. In considering which foods to add to the eFFQ’s food list, foods that were sporadically mentioned in the 24HR, i.e., accounting for only 5% or less of the total foods mentioned, were excluded [35]. The data from these 24HRs served as the basis for selecting regional foods and in describing portion sizes using familiar or commonly used kitchen/serving utensils. Participants in this survey were not included in the final sample of 163 participants for the eFFQ validation.

The semiquantitative eFFQ consists of a 76-item food list, a frequency of intake section (every day, every week, every month, or no consumption), a portion size section, and the number of servings consumed (0–14 servings) section. Foods are grouped under the following 10 categories: vegetables; fruits; bread, grains, and starches; legumes and derivatives; dairy products and derivatives; eggs, meats, fish, and derivatives; fats, oils, and nuts; sugar and sweets; condiments, dressings, and spices; and beverages and infusions. Also, where appropriate, items include options for foods with 100% plant food ingredients. The eFFQ asks respondents to indicate how often they consume the foods, the usual portion size eaten, and the number of servings every time they ate that food during the past 12 months (see Figure S1). The reported consumption of each food item was converted to grams per day for evaluation.

### 2.5. Nutrient Intake Determination

Dietary intake reports on both the eFFQ and 24HR were coded using the MAR24 tool [33]. The nutrient profile of food items was derived from the USDA Food Composition Databases [36], except for 5 particular foods (Amargo serrano, Amargo serrano diet, Bizcochos de grasa, Chipá, and pan criollo) for which the composition was obtained from the Argentinian Food Registry System (SARA) [37]. Each food item selected from the USDA database [36] had its nutrient composition (water, energy, fiber, macro, and micronutrients) and ingredient/s (when applicable) compared and checked for similarity with data from the following available Argentinian databases: SARA, ArgenFood Food Composition Table, Nutrinfo database, or the Central American Food Composition Table of the Institute of Nutrition of Central America and Panama (INCAP) [38]. For cooked foods, all cooking methods were searched for each food and those used in Argentina were selected. As there is no information on cooked foods from local databases for comparison, cooked versions of the raw foods from the same USDA data source (Legacy, Survey, or Foundation) were selected [36]. 

### 2.6. Statistical Analysis

Descriptive statistics were performed to report the means and standard deviations for energy and nutrient intake variables that were normally distributed, and medians and interquartile ranges for variables with non-normal distributions. These were conducted for the first eFFQ administration (eFFQ1), second eFFQ administration (eFFQ2), and the 24HR. Energy adjustment was performed using the residual method proposed by Willett et al. [39].

#### 2.6.1. Validation of the eFFQ 

The ability of our eFFQ to discriminate among individuals and measure true dietary intake was evaluated by comparing individual estimates of nutrient intake based on the questionnaire with those measured by a more accurate method, considered as a gold standard (relative validity). The primary alternative to the use of diet records as a standard for evaluating an FFQ is the collection of the 24HR [17,18,19,20,29]. Although this method relies on memory and the perception of serving sizes, it is considered an option in situations where subjects are illiterate [15] or less highly motivated, such as individuals not experienced in participating in scientific studies. Hence, to determine the relative validity of the eFFQ, the eFFQ2—which was administered after the four 24HRs were collected—was compared with the reference method, the 24HR. The differences in the reported intakes between the methods were calculated using either the ^t^-test or the Wilcoxon signed-rank test, depending on the distribution of the variables. The Spearman’s and Pearson’s correlation coefficients were calculated for crude, energy-adjusted, and de-attenuated data. The de-attenuation process of the correlations to correct for within-person variation in multiple 24HRs was performed with the method using the PC-SIDE software (Version 1.0, software for intake distribution estimation for the Windows operating system) which was developed by researchers in the statistics department of Iowa State University [40]. The ranking ability of the eFFQ was determined with cross-classification [41]. Cross-classification was accomplished by ranking the intake values for both the mean of the four 24HRs and eFFQ into quintiles and then cross-tabulating the quintiles to determine the agreement and gross misclassification in reported intakes between the measures. The proportions of respondents with exact agreement (EA) on the quintile classifications of their reported intake in both methods, those whose intakes deviated from exact agreement by one (EA ± 1) or two (EA ± 2) quintiles, and those whose intakes were on opposite quintiles (grossly misclassified) were computed. We calculated the weighted kappa statistic for the ranking. Finally, Bland–Altman plots [42] were graphed for macronutrients to further determine the agreement between the two methods and evaluate outliers or possible biases. 

#### 2.6.2. Reproducibility of the eFFQ

To determine the reproducibility of the eFFQ, we calculated the intraclass correlations (ICC) for unadjusted/crude and energy-adjusted values between eFFQ1 and eFFQ2. Ranking by quintiles and cross-classification were performed to determine the level of agreement and gross misclassification between the two eFFQ administrations. The weighted kappa statistic was also calculated. All statistical values were considered significant when *p* < 0.05.

## 3. Results

### 3.1. Demographic Profile of Participants

Among 186 subjects initially enrolled in the study, 23 either did not fully complete the questionnaires, had changed their usual diet for medical or other reasons during the past year, or reported an energy intake outside the range of 500–4000 kcal. These were excluded from the analysis [31], and the analytical data were based on 163 participants. 

Table 1 shows the demographic characteristics of the study population. Approximately 85% of the participants were females, and about 28% belonged to the Buenos Aires region. About 38% had a tertiary level of education and about 88% were employees. The mean age was 41.8 ± 10.4 years and the mean body mass index (BMI) was 26.7 ± 4.9 kg/m^2^.

### 3.2. Validity of the eFFQ Nutrient and Bioactive Compound Estimates

Data from the eFFQ2 were used to determine the relative validity of the eFFQ since this covers the 24HR data collection period. Compared to the 24HR, both the crude and energy-adjusted nutrient intake estimates of the eFFQ were higher for most of the nutrients, except for total sugar, lycopene, and caffeine (see Table 2). Comparison tests indicated that the intakes of energy and most nutrients, except for cholesterol, β cryptoxanthin, ethanol, and theobromine, were significantly different. Table 2 shows the crude, energy-adjusted, and de-attenuated correlation coefficients between the eFFQ and the 24HR, with crude correlation coefficients ranging from 0.15 for phosphorous and niacin to 0.57 for caffeine; most were within an acceptable range. Energy-adjustment slightly increased the correlations, ranging from 0.15 for vitamin E to 0.58 for caffeine, while the de-attenuated correlations ranged from 0.16 for niacin to 0.57 for animal protein.

The levels of agreement are also shown in Table 2. The proportions of agreement within one quintile adjacent to exact agreement (EA ± 1) ranged from 52.2% and around 74% for linoleic acid (LA) and caffeine, respectively. The levels of agreement for those within two adjacent quintiles of exact agreement (EA ± 2) ranged from 77.9% for ethanol to 93.3% for animal protein and magnesium. Extreme misclassifications into the opposite quintiles were less than 7% for energy and all nutrients. The weighted kappa values described conformity ranging from 0.04 (LA) to 0.38 (caffeine) between eFFQ2 and the mean of 24HR.

**Figure 2:**
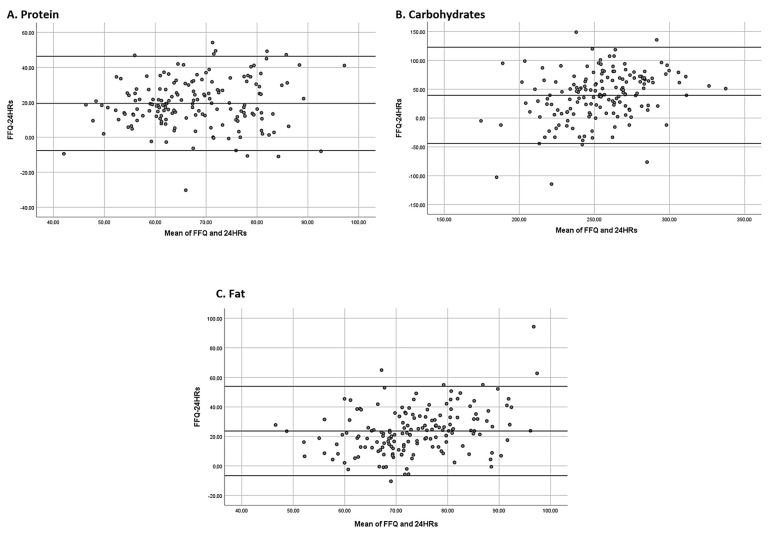
Bland–Altman graphs after plotting the mean differences between the eFFQ and the 24HR against the product of the means of the eFFQ and 24HR on estimates of macronutrient intakes.

The Bland–Altman plots (see Figure 2) show that macronutrient intakes were overestimated by the eFFQ. 

### 3.3. Reproducibility of the eFFQ 

Table 3 shows the crude and energy-adjusted intraclass correlation coefficient (ICC) for eFFQ1 and eFFQ2. The crude ICC ranged from 0.29 to 0.85 for lycopene and ethyl alcohol, respectively. However, the correlation coefficients differed slightly after adjusting for energy. The energy-adjusted ICC ranged from 0.23 for lycopene to 0.89 for magnesium. The ICC shows moderate and good correlations for most nutrients except for alpha and beta-carotene, lycopene, lutein zeaxanthin, vitamins A, C, and E for unadjusted values, and vitamin A, alpha-carotene, beta-carotene, and lycopene for energy-adjusted ICC values. In cross-classification analysis, the ranges of the agreement levels for the EA ± 1 quintile were from 70.6% for water, choline, and vitamin E to 87.7% for caffeine. Extreme misclassification into the opposite quintiles was less than 5% for energy and all nutrients. The weighted kappa values described fair and moderate conformity, ranging from 0.21 (vitamin B12) to 0.62 (eicosapentaenoic acid) between the two administrations of the eFFQ.

## 4. Discussion

This work investigated the validity and reproducibility of an electronic food frequency questionnaire (eFFQ) in estimating the intake of nutrients and bioactive compounds of an adult population from the six main regions of Argentina. The results show that this eFFQ is relatively valid in ranking adults according to their nutrient intake and has an acceptable reproducibility. However, it slightly overestimates the intake of most nutrients. 

In Argentina, only a few published studies have evaluated the reproducibility and validity of an FFQ used in the adult population. Navarro et al. conducted a study with 66 individuals 23–80 years of age to validate a specific FFQ for adults with cancer in Córdoba, Argentina [29]. In 2012, Dehghan et al. validated an FFQ with 116 women and 40 men residing in rural and urban areas of Rosario [43]. Elorriaga et al. evaluated the dietary intake of 147 individuals 21–74 years of age from the southern cone of South America (Argentina, Uruguay, and Chile) [18]. Zapata and colleagues validated an FFQ with a sample of 88 adults from Rosario, Argentina [19]. Perovic et al. validated an FFQ to assess the intake of lipids and phytochemicals on a sample of 45 individuals of both sexes, aged between 20 and 72 years [17]. Finally, Olmedo et al. validated a questionnaire to estimate the intake of free sugars and ultra-processed foods of 77 residents of La Plata city and its surroundings [44]. These studies were conducted in specific areas in Argentina, but none considered sampling from the different regions of the country. To the best of our knowledge, this is the first study that validated and tested the reproducibility of an FFQ that was developed in electronic format to estimate and rank the dietary intake of an adult population from the six main regions of Argentina. A total of 163 subjects from different regions of the country participated in this study. Sample size in FFQ validations is variable but, in most studies, it has been less than 200 individuals [18,19,45,46,47,48,49]. Bland–Altman graphs were used to evaluate the absolute concordance between FFQ and 24HR, which recommends using a sample of at least 50 and preferably 100 participants [43]. 

The number of food items in an FFQ tends to vary widely, ranging from 5 to 350, with an average of 79 questions [50], which is similar to our eFFQ. There is a rapidly diminishing marginal gain in the information obtained from increasingly detailed questionnaires [51,52,53]. Therefore, a long list of food items is not recommended because of the tendency for the overestimation of reported intake in FFQ [54]. The FFQs validated in Argentinian adult populations vary from 66 to 257 food items [17,19]. This present eFFQ has a total of 76 items grouped into 10 food groups, which cover the main foods consumed in Argentina. It is important to highlight that, despite regional variations, Argentine food intake presents a certain monotony; thus, a comprehensive FFQ such as ours may sufficiently cover the foods currently eaten in the different regions of the country. 

To assess the reproducibility of the questionnaire, we compared the eFFQ data on two time points. Time intervals between the repeated administration of FFQs range from a few days [55,56,57] to more than one year [58]. For better results, some authors recommend a period of 4 to 6 months between the repeated administration of an FFQ [51,59]. Closer repeated administrations of FFQs allow high correlations, probably because subjects may still remember their previous responses. On the other hand, a long interval may result in weak correlations for variations in responses due to actual changes and disturbances in dietary intake during the time between the two administrations [58,60]. In our study, our repeat administration of the eFFQ was approximately 12 months apart from the first administration which could explain the lower correlation coefficients for reproducibility. According to Willet and Lenart [61], this limitation should not be considered serious from the point of view of measurement error. The mean intakes of most nutrients from our eFFQ1 were higher compared to the data obtained from eFFQ2. This may be due to a training effect, where intake reporting has improved, or due to a real change in dietary intake.

The crude ICCs for the reproducibility of our eFFQ ranged between 0.29 and 0.85, and these results are similar to other studies [60,62]. After adjusting for energy, the ICC for most of the nutrient estimates changed, where most of the coefficients increased while some decreased, with results ranging from 0.23 to 0.89. The association between nutrient intake and energy intake may cause the correlation coefficients to increase after energy adjustment. On the other hand, the decreased correlation coefficients after energy adjustment could be due to systematic overestimation or underestimation [63]. In the cross-classification analysis, the ranges of concordance levels for the same category or in an adjacent category between eFFQ1 and eFFQ2 ranged between 70.6% and 87.7%, similar to another study [60]. The extreme misclassification in the opposite quintiles was <4.9% for energy and all nutrients. 

In testing the validity of the eFFQ, crude correlations between eFFQ2 and the mean of multiple 24HR ranged from 0.15 to 0.57, which increased slightly after energy adjustment (0.15–0.58). Our crude correlations were similar to a study among urban participants by Dehghan et al. [43] with coefficients of 0.20–0.47, while the correlations after energy adjustment were similar to those of Ye et al. [64] with energy-adjusted correlations of 0.19 to 0.58. In our cross-classification analyses, the agreements within one quintile between the eFFQ and 24HR ranged from approximately 63% to 72% for macronutrients and approximately 52% to 73% for micronutrients. These results are very similar to another study, where values ranged between 50% and 75% for macronutrients and 48% and 70% for micronutrients [65]. Extreme misclassification was less than 7% for energy and all nutrients; similar findings were observed in a study conducted in Indonesia [66]. On the other hand, the weighted kappa values between eFFQ2 and the mean of 24HR showed a compliance that ranged from 0.04 to 0.38, results similar to the study carried out by Zhuang et al. [60] whose values ranged between 0.12 and 0.40. 

Argentina is a country whose diet is largely focused on the consumption of meat, which is the main source of protein. National statistics show that 5 out of 10 individuals consume red meat, poultry, and/or eggs at least once a day [67]. On the other hand, in 2021, Argentina was the country with the highest consumption of beef per inhabitant on the planet, while it ranks ninth in the consumption of chicken meat [68]. Our eFFQ is able to aptly rank individuals on their intake of animal protein as indicated by its moderately strong de-attenuated correlation (r = 0.57) with the reference standard. 

The USDA database, which is widely comprehensive in terms of micro- and macronutrient information, was used with the MAR24 tool, an available resource developed for 24HR and nutrient intake estimation in Argentina. The lack of comprehensive nutrient information from Argentine food composition tables is a limitation, but having the USDA database provides security since its data are highly reliable [36]. In many resource-deficient areas of the world where food composition databases are incomplete, outdated, or lacking, the use and sharing of existing databases has become standard practice [69]. Energy intake estimates with our eFFQ could have been validated against doubly labeled water, but this was not feasible in our context. This is often a limitation in many validation studies with limited resources. One limitation of the study is that the study participants were not randomly chosen from the general population but were volunteers who met the study inclusion criteria. However, in general, validation studies are carried out among voluntary participants [70]. Another limitation of the study is the low percentage of male participants (15.3%). 

The lack of a more objective reference method, such as the use of biomarkers to triangulate the validation of the eFFQ, is a limitation. However, about 75% of the methods used for the validation of FFQs employ as a reference method 24HR and dietary records [50]. We chose unannounced non-consecutive 24HR as the reference method to validate our eFFQ since this will prevent respondents from meal planning beforehand to report an intake that sounds desirable to the interviewer. Although the 24HR has correlated errors with FFQ due to reliance on memory, recalling the immediate past such as in 24HR employs episodic memory whereas generic memory is what is used in FFQs when recalling the usual intake of the past [16,71]. Multi-day weighed food recording would have eliminated the errors associated with the recall, but participants may (un)consciously alter their food intake to ease the burden associated with the tedious process of food recording [51]. However, it should be noted that food intake cannot be estimated without error, and the nature and magnitude of the error are related not only to the method selected to collect the data but also to the subjects studied [72]. 

## 5. Conclusions

This is the first study that validated an instrument developed for the adult population living in different regions of Argentina. As an online tool, the eFFQ allows ease in the administration of the instrument, facilitating its use in population studies. If self-administered, respondents may feel more at ease in filling out the questionnaire in the convenience of their own home or time with a greater assurance of confidentiality. The results of this study show that this eFFQ is relatively valid in ranking adults according to their nutrient intake and has an acceptable reproducibility, yet it slightly overestimates the intake of most nutrients.

## Figures and Tables

**Table 1:** Demographic characteristics of the study population.

Characteristics	Distribution			
	n	Mean	%	SD
Gender				
Male	25		15.3	
Female	138		84.7	
Age		41.8		10.4
Marital status				
Currently married	124		76.1	
Married in the past	12		7.4	
Never married	27		16.6	
Region of Argentina				
Buenos Aires	45		27.6	
Central	32		19.6	
Cuyo	26		16.0	
Northeast	21		12.9	
Northwest	17		10.4	
South	22		13.5	
Education level				
Secondary school or less	35		21.5	
Tertiary	62		38.0	
University	61		37.4	
Graduate school	5		3.1	
Occupational status				
Unemployed	1		0.6	
Employed	143		87.7	
Retired	2		1.2	
Unpaid domestic work	11		6.7	
Independent worker	6		3.7	
BMI (kg/m^2^)		26.7		4.9
<18.5	2		1.2	
18.5–24.9	64		39.3	
25–29.9	58		35.6	
≥30	39		23.9	

**Table 2:** Relative validity of the eFFQ. Comparisons, correlations, and agreement between the eFFQ and the 24HR reference standard.

Nutrient	24HR				eFFQ ^a^				Correlations			% Agreement (24HR vs. eFFQ) by Quintile				Weighted Kappa
	Crude		Energy-Adjusted		Crude		Energy-Adjusted									
	Median	IQR	Median	IQR	Median	IQR	Median	IQR	Crude	Energy-Adjusted	De-Attenuated	Within Same Quintile	±1 Quintile	±2 Quintiles	Grossly Misclassified	
Energy (kcal)	1680.2	540.0			2069.7	965.3			0.20 **			28.2	57.7	83.4	4.3	0.15 *
Water (g)	2270.6	958.9	2252.3	936.0	2346.5	1116.7	2439.6	992.9	0.09	0.40 **	0.39 **	30.1	66.3	85.3	3.1	0.24 **
Total protein (g)	57.6	20.2	56.8	16.2	75.6	37.9	76.7	17.0	0.18 *	0.44 **	0.37 **	28.8	69.9	90.2	3.1	0.28 **
Animal protein (g)	32.9	24.2	31.4	21.7	38.8	30.4	37.3	26.3	0.46 **	0.54 **	0.57 **	34.4	72.4	93.3	0.6	0.37 **
Vegetable protein (g)	24.8	11.4	24.5	8.7	37.4	19.4	36.8	13.5	0.30 **	0.45 **	0.45 **	31.9	66.3	90.8	1.8	0.29 **
Carbohydrate (g)	232.3	84.3	235.5	36.6	270.7	128.3	275.2	53.2	0.22 **	0.27 **	0.28 **	26.4	63.8	85.3	5.5	0.18 *
Dietary fiber (g)	28.0	17.5	28.3	14.6	33.2	18.5	32.3	13.6	0.34 **	0.43 **	0.50 **	29.5	68.7	89.0	3.7	0.27 **
Total sugar (g)	102.7	41.6	101.8	31.3	96.8	50.0	93.3	27.4	0.40 **	0.35 **	0.40 **	31.9	62.6	83.4	1.8	0.22 **
Fat (g)	62.7	29.8	61.7	12.2	83.1	47.0	84.7	18.3	0.24 **	0.25 **	0.26 **	30.1	59.5	84.1	4.9	0.17 *
SFA (g)	20.4	12.3	20.7	6.1	26.2	15.2	25.1	8.5	0.36 **	0.35 **	0.39 **	25.2	59.5	87.7	0.0	0.20 **
MUFA (g)	19.5	10.3	19.2	4.9	26.4	16.0	27.2	6.9	0.20 **	0.27 **	0.23 **	25.2	57.1	83.4	4.3	0.13 *
PUFA (g)	15.3	9.3	15.3	6.5	23.3	13.6	23.6	8.0	0.10	0.07	0.07	20.3	54.0	79.8	4.9	0.05
LA 18:2n-6 (g)	13.8	8.9	13.8	6.4	20.6	12.9	21.0	7.5	0.10	0.23 **	0.22 **	21.5	52.2	79.1	4.9	0.04
ALA 18:3n-3 (g)	0.8	0.6	0.8	0.4	1.8	1.1	1.7	0.9	0.17 *	0.23 **	0.22 **	23.3	61.4	82.2	3.1	0.14 *
EPA 20:5n-3 (g)	0.00	0.01	0.00	0.01	0.02	0.02	0.02	0.02	0.28 **	0.29 **	0.31 **	27.0	60.1	87.7	3.7	0.19 **
DPA 22:5n-3 (g)	0.01	0.02	0.01	0.02	0.02	0.02	0.02	0.02	0.46 **	0.52 **	0.52 **	33.7	72.4	92.0	1.2	0.35 **
DHA 22:6n-3 (g)	0.03	0.04	0.03	0.04	0.06	0.06	0.06	0.06	0.32 **	0.33 **	0.33 **	29.5	65.0	85.3	3.7	0.22 **
Cholesterol (mg)	210.2	126.3	220.6	127.8	220.8	142.5	214.3	110.7	0.28 **	0.26 **	0.34 **	25.8	60.1	85.3	4.9	0.16 *
Thiamine (mg)	1.3	0.5	1.3	0.3	1.7	0.9	1.7	0.5	0.21 **	0.35 **	0.34 **	23.3	62.0	87.7	1.8	0.19 **
Riboflavin (mg)	1.6	0.6	1.6	0.4	2.0	1.0	2.0	0.6	0.28 **	0.41 **	0.42 **	27.0	69.3	89.6	3.7	0.26 **
Niacin (mg)	14.3	5.6	14.8	4.3	17.8	8.6	17.7	3.8	0.15 *	0.18 *	0.16 *	23.9	61.4	85.3	4.3	0.16 *
Pantothenic acid (mg)	1.9	0.9	2.0	0.8	2.3	1.1	2.3	0.6	0.08	0.13	0.19 *	20.3	55.2	79.8	6.1	0.05
Pyridoxine (mg)	1.4	0.7	1.4	0.5	2.0	1.0	2.0	0.7	0.29 **	0.38 **	0.38 **	26.4	67.5	88.3	1.2	0.25 **
Folic acid (µg)	284.3	153.1	289.7	108.3	413.9	181.7	403.0	137.0	0.27 **	0.48 **	0.53 **	32.5	67.5	89.6	0.0	0.31 **
Choline (mg)	213.5	105.3	212.2	85.9	245.5	115.3	248.5	65.6	0.20 **	0.30 **	0.34 **	28.2	60.7	85.9	3.7	0.19 **
Vitamin B12 (µg)	2.5	2.0	2.4	1.8	3.3	2.2	3.2	1.8	0.30 **	0.34 **	0.37 **	28.2	65.0	87.1	3.1	0.23 **
Vitamin C (mg)	82.2	79.5	81.6	82.2	108.4	90.9	103.1	80.0	0.39 **	0.39 **	0.39 **	33.7	66.3	87.7	3.1	0.28 **
Vitamin A (µg RAE)	532.5	370.9	547.7	352.6	803.2	427.8	784.6	378.5	0.15	0.20 **	0.18 *	26.4	62.0	81.0	5.5	0.14 *
Retinol (µg)	229.9	154.1	225.1	119.2	298.6	204.2	292.9	150.2	0.26 **	0.28 **	0.28 **	27.0	62.6	81.0	4.3	0.16 *
Vitamin E (mg)	9.0	4.8	8.8	4.0	12.4	6.9	12.9	4.4	0.14	0.15 *	0.17 *	28.8	58.3	81.0	6.8	0.13 *
Vitamin D (IU)	77.3	75.6	77.3	75.6	116.4	119.6	116.7	93.3	0.32 **	0.31 **	0.35 **	24.5	61.4	86.5	2.5	0.18 *
Vitamin K (µg)	81.6	135.3	73.3	125.3	162.5	115.2	153.1	105.5	0.23 **	0.27 **	0.40 **	22.1	64.4	85.9	3.7	0.17 *
Ca (mg)	770.4	391.8	760.7	306.5	1031.9	538.0	975.1	329.7	0.24 **	0.27 **	0.26 **	22.7	63.2	84.7	3.1	0.17 *
Fe (mg)	12.0	4.5	12.0	2.8	15.7	7.4	15.6	3.9	0.17 *	0.24 **	0.25 **	30.7	59.5	81.6	3.1	0.17 *
Mg (mg)	256.0	115.5	254.4	87.8	353.9	171.1	345.3	136.7	0.39 **	0.55 **	0.53 **	34.4	71.8	93.3	2.5	0.35 **
P (mg)	862.0	327.5	862.3	155.6	1317.1	658.6	1290.6	302.0	0.15 *	0.25 **	0.24 **	23.9	60.1	82.8	4.3	0.14 *
K (mg)	2571.6	922.7	2571.0	725.1	3213.2	1612.8	3229.6	1294.0	0.38 **	0.45 **	0.46 **	31.3	70.6	87.1	0.6	0.30 **
Na (mg)	1824.7	870.4	1839.9	607.7	2517.1	1251.2	2536.4	809.0	0.23 **	0.32 **	0.35 **	27.0	68.7	83.4	4.3	0.21 **
Zn (mg)	7.5	3.7	7.3	2.8	10.4	5.3	10.6	2.6	0.13	0.19 *	0.19 *	22.1	59.5	84.7	6.1	0.12 *
Cu (mg)	1.2	0.5	1.2	0.4	1.6	0.8	1.6	0.6	0.36 **	0.51 **	0.52 **	35.0	68.7	92.0	1.2	0.34 **
Mn (mg)	1.4	0.9	1.4	1.0	2.4	1.7	2.4	1.6	0.30 **	0.36 **	0.37 **	34.4	65.6	89.6	2.5	0.29 **
Se (mg)	66.6	28.5	66.0	25.5	97.6	51.0	98.2	28.3	0.19 *	0.29 **	0.36 **	27.0	62.6	85.9	6.8	0.17 *
β carotene (µg)	2806.7	3349.2	2829.8	3347.2	4475.2	4025.0	4448.8	4082.4	0.23 **	0.25 **	0.28 **	32.5	59.5	82.2	5.5	0.17 *
α carotene (µg)	664.6	1212.6	666.4	1188.6	1457.6	1719.1	1425.1	1572.7	0.20 *	0.21 **	0.20 **	26.4	62.6	84.1	6.8	0.16 *
β cryptoxanthin (µg)	197.3	280.6	193.8	302.6	229.5	223.6	223.9	190.3	0.43 **	0.43 **	0.49 **	30.7	68.1	90.2	1.8	0.29 **
Lycopene (µg)	2264.1	2422.7	2270.9	2274.5	1673.6	1046.2	1661.8	975.6	0.19 *	0.24 **	0.24 **	25.8	63.2	81.6	4.3	0.16 *
Lutein + zeaxanthin (µg)	1457.7	3302.1	1354.9	3651.5	2742.8	2306.6	2656.1	2229.8	0.21 **	0.23 **	0.34 **	22.7	57.1	85.3	3.7	0.13 *
Ethanol (g)	0.0	0.0	0.0	0.0	0.0	0.0	0.0	0.0	0.12	0.23	0.07	27.0	60.1	77.9	14.7	0.09
Caffeine (mg)	16.3	37.0	16.3	37.0	12.0	20.3	11.1	18.9	0.57 **	0.58 **	0.52 **	36.2	73.6	92.6	0.6	0.38 **
Theobromine (mg)	14.7	45.0	14.7	45.0	18.0	21.7	18.0	21.7	0.30 **	0.31 **	0.17	24.5	58.3	82.8	3.7	0.17 *

24HR = 24 h dietary recall; eFFQ = electronic food frequency questionnaire; SFA = saturated fatty acid; MUFA = monounsaturated fatty acid; PUFA = polyunsaturated fatty acid; LA = linoleic acid; ALA = alpha-linolenic acid; EPA = eicosapentaenoic acid; DPA = docosapentaenoic acid; DHA = docosahexaenoic acid; RAE = retinol activity equivalent. IQR = interquartile range. ^a^ second administration of the FFQ, i.e., FFQ2, was used to determine validity of the nutrient intake estimates. * *p* < 0.05; ** *p* < 0.001.

**Table 3:** Reproducibility of the eFFQ. Comparisons, correlations, and agreement between eFFQ1 and eFFQ2.

Nutrient	eFFQ1				eFFQ2				ICC		% Agreement by Quintile				Weighted Kappa
	Crude		Energy-Adjusted		Crude		Energy-Adjusted								
	Median	IQR	Median	IQR	Median	IQR	Median	IQR	Unadjusted	Energy-Adjusted	Within Same Quintile	±1 Quintile	±2 Quintiles	Grossly Misclassified	
Energy (kcal)	2127.3	922.2			2069.7	965.3			0.62 **		36.8	76.7	91.4	4.3	0.38 ***
Water (g)	2390.7	1075.6	2353.6	1080.7	2346.5	1116.7	2439.6	992.9	0.63 **	0.63 **	39.9	70.6	90.2	3.7	0.35 ***
Total protein (g)	76.7	37.0	78.7	20.7	75.6	37.9	76.7	17.0	0.64 **	0.71 **	44.2	79.1	92.6	0.6	0.47 ***
Animal protein (g)	41.3	30.3	41.8	32.1	38.8	30.4	37.3	26.3	0.74 **	0.82 **	42.9	83.4	96.9	0.6	0.51 ***
Vegetable protein (g)	35.7	23.9	35.5	15.0	37.4	19.4	36.8	13.5	0.72 **	0.85 **	44.8	80.4	96.9	0.6	0.51 ***
Carbohydrate (g)	267.7	132.6	280.7	46.6	270.7	128.3	275.2	53.2	0.62 **	0.68 **	36.2	75.5	90.8	1.8	0.38 ***
Fiber (g)	30.7	20.1	30.9	14.8	33.2	18.5	32.3	13.6	0.70 **	0.84 **	41.7	81.6	94.5	0.0	0.48 ***
Total sugar (g)	97.4	56.5	97.6	32.8	96.8	50.0	93.3	27.4	0.61 **	0.64 **	33.7	74.9	92.0	2.5	0.36 ***
Fat (g)	87.2	43.0	88.8	17.8	83.1	47.0	84.7	18.3	0.60 **	0.66 **	35.6	76.1	92.0	0.6	0.39 ***
SFA (g)	26.5	16.2	27.1	10.0	26.2	15.2	25.1	8.5	0.61 **	0.73 **	42.3	79.8	94.5	0.0	0.36 ***
MUFA (g)	28.2	14.3	28.1	7.1	26.4	16.0	27.2	6.9	0.62 **	0.65 **	33.1	74.2	92.0	1.2	0.30 ***
PUFA (g)	24.8	13.4	23.9	7.9	23.3	13.6	23.6	8.0	0.52 **	0.58 **	29.5	72.4	89.6	3.1	0.42 ***
LA 18:2n-6 (g)	22.3	11.9	21.1	7.4	20.6	12.9	21.0	7.5	0.64 **	0.70 **	38.0	76.1	94.5	0.6	0.58 ***
ALA 18:3n-3 (g)	1.84	1.48	1.82	0.94	1.77	1.11	1.72	0.90	0.81 **	0.82 **	48.5	85.9	98.2	0.0	0.58 ***
EPA 20:5n-3 (g)	0.02	0.02	0.02	0.02	0.02	0.02	0.02	0.02	0.81 **	0.83 **	49.1	86.5	96.9	0.0	0.62 ***
DPA 22:5n-3 (g)	0.02	0.02	0.02	0.02	0.02	0.02	0.02	0.02	0.82 **	0.84 **	56.4	85.9	97.6	0.0	0.29 ***
DHA 22:6n-3 (g)	0.07	0.06	0.06	0.06	0.06	0.06	0.06	0.06	0.50 **	0.56 **	28.8	73.6	88.3	3.7	0.46 ***
Cholesterol (mg)	228.8	138.5	230.2	117.4	220.8	142.5	214.3	110.7	0.75 **	0.82 **	42.3	77.3	94.5	0.0	0.31 ***
Thiamine (mg)	1.7	1.0	1.7	0.5	1.7	0.9	1.7	0.5	0.64 **	0.64 **	37.4	75.5	91.4	3.7	0.41 ***
Riboflavin (mg)	2.0	1.0	2.0	0.7	2.0	1.0	2.0	0.6	0.56 **	0.59 **	39.9	75.5	93.3	1.8	0.43 ***
Niacin (mg)	17.9	8.5	18.0	5.1	17.8	8.6	17.7	3.8	0.69 **	0.67 **	38.7	78.5	94.5	2.5	0.37 ***
Pantothenic acid (mg)	2.3	0.9	2.3	0.6	2.3	1.1	2.3	0.6	0.62 **	0.70 **	36.2	74.2	90.2	1.2	0.44 ***
Pyridoxine (mg)	1.9	0.9	2.0	0.8	2.0	1.0	2.0	0.7	0.56 **	0.67 **	42.9	77.3	92.0	1.8	0.45 ***
Folic acid (µg)	408.9	208.6	410.7	158.6	413.9	181.7	403.0	137.0	0.68 **	0.77 **	42.9	77.9	93.3	1.2	0.37 ***
Choline (mg)	249.0	94.8	241.4	60.6	245.5	115.3	248.5	65.6	0.61 **	0.72 **	38.0	70.6	92.0	1.2	0.46 ***
Vitamin B12 (µg)	3.3	2.2	3.2	2.3	3.3	2.2	3.2	1.8	0.71 **	0.77 **	45.4	74.2	95.1	0.6	0.21 ***
Vitamin C (mg)	105.3	92.0	108.4	82.8	108.4	90.9	103.1	80.0	0.41 **	0.60 **	39.3	71.2	90.2	3.7	0.38 ***
Vitamin A (µg RAE)	840.2	483.3	797.7	321.8	803.2	427.8	784.6	378.5	0.37 **	0.47 **	28.8	65.6	85.3	4.9	0.34 ***
Retinol (µg)	311.3	223.0	313.9	180.2	298.6	204.2	292.9	150.2	0.60 **	0.71 **	31.9	74.2	92.0	2.5	0.34 ***
Vitamin E (mg)	13.4	6.5	13.0	4.3	12.4	6.9	12.9	4.4	0.45 **	0.56 **	28.2	70.6	92.0	1.2	0.51 ***
Vitamin D (IU)	119.1	125.8	118.2	101.8	116.4	119.6	116.7	93.3	0.62 **	0.75 **	43.6	85.3	95.1	1.2	0.35 ***
Vitamin K (µg)	156.9	134.2	161.1	124.3	162.5	115.2	153.1	105.5	0.51 **	0.51 **	39.9	76.7	90.8	3.1	0.48 ***
Ca (mg)	1047.3	542.7	1046.0	396.3	1031.9	538.0	975.1	329.7	0.52 **	0.61 **	34.4	68.7	87.7	2.5	0.36 ***
Fe (mg)	15.6	6.1	15.6	4.1	15.7	7.4	15.6	3.9	0.63 **	0.58 **	33.1	74.9	93.3	3.1	0.60 ***
Mg (mg)	347.1	190.9	348.8	125.3	353.9	171.1	345.3	136.7	0.74 **	0.89 **	52.2	86.5	97.6	0.0	0.40 ***
P (mg)	1329.7	595.0	1318.1	282.2	1317.1	658.6	1290.6	302.0	0.60 **	0.67 **	38.7	74.9	92.6	1.8	0.41 ***
K (mg)	3315.0	1529.2	3312.8	1209.0	3213.2	1612.8	3229.6	1294.0	0.50 **	0.60 **	38.7	76.7	91.4	1.2	0.34 ***
Na (mg)	2672.2	1426.9	2641.6	773.3	2517.1	1251.2	2536.4	809.0	0.53 **	0.51 **	38.0	71.2	89.0	3.7	0.40 ***
Zn (mg)	10.7	4.7	10.9	2.4	10.4	5.3	10.6	2.6	0.64 **	0.61 **	37.4	78.5	90.2	1.8	0.49 ***
Cu (mg)	1.6	0.9	1.6	0.6	1.6	0.8	1.6	0.6	0.71 **	0.88 **	40.5	81.0	97.6	0.0	0.52 ***
Mn (mg)	2.1	1.5	2.1	1.3	2.4	1.7	2.4	1.6	0.73 **	0.83 **	42.9	85.3	96.3	0.6	0.36 ***
Se (mg)	99.1	43.4	99.4	27.3	97.6	51.0	98.2	28.3	0.70 **	0.69 **	35.0	73.0	91.4	1.2	0.35 ***
β carotene (µg)	4514.6	4245.8	4234.7	3329.7	4475.2	4025.0	4448.8	4082.4	0.41 **	0.45 **	38.7	68.7	89.0	1.8	0.27 ***
α carotene (µg)	1377.1	1588.2	1334.4	1210.9	1457.6	1719.1	1425.1	1572.7	0.37 **	0.39 **	31.9	69.9	85.9	4.3	0.34 ***
β cryptoxanthin (µg)	212.0	241.1	212.5	220.8	229.5	223.6	223.9	190.3	0.51 **	0.61 **	35.0	72.4	90.2	1.8	0.29 ***
Lycopene (µg)	1822.0	1324.8	1726.3	1047.1	1673.6	1046.2	1661.8	975.6	0.29 *	0.23 *	33.1	69.9	88.3	4.3	0.38
Lutein + zeaxanthin (µg)	2663.9	2292.1	2644.3	2059.8	2742.8	2306.6	2656.1	2229.8	0.44 **	0.51 **	37.4	74.9	90.2	1.8	0.31 ***
Ethyl alcohol (g)~	0.0	0.0	0.0	0.0	0.0	0.0	0.0	0.0	0.85 **	0.85 **	39.3	79.8	93.3	1.8	0.43 ***
Caffeine (mg)	12.1	23.0	12.4	23.0	12.0	20.3	11.1	18.9	0.83 **	0.81 **	51.5	87.7	95.7	1.2	0.54 ***
Theobromine (mg)~	19.6	32.9	19.6	32.9	18.0	21.7	18.0	21.7	0.80 **	0.80 **	42.9	80.4	96.3	0.6	0.49 ***

SFA = saturated fatty acid; MUFA = monounsaturated fatty acid; PUFA = polyunsaturated fatty acid; LA = linoleic acid; ALA = alpha-linolenic acid; EPA = eicosapentaenoic acid; DPA = docosapentaenoic acid; DHA = docosahexaenoic acid; IQR = interquartile range; ICC = interclass correlation coefficient. ~ Nutrients that are not adjusted for energy. * *p* < 0.05; ** *p* < 0.01; *** *p* < 0.0001.

## Data Availability

The data presented in this study are available on request from the corresponding author. The data are not publicly available due to privacy.

## Supplementary Materials

The following supporting information can be downloaded at: https://www.mdpi.com/article/10.3390/nu16111564/s1, Figure S1: eFFQ question example.
