# Supplementary material for: Validity and Reproducibility of an Electronic Food Frequency Questionnaire in Argentinian Adults
Source: Nutrients. 2024 May 22;16(11):1564. doi: 10.3390/nu16111564 (PMC11174462; doi:10.3390/nu16111564)

Validity and Reproducibility of an Electronic Food Frequency Questionnaire in Argentinian Adults

Rocio Victoria Gili [1,2,*], Sara Leeson [1], Belén Carlino [1,2], Ismael Alejandro Contreras-Guillén [1,3], Daniel Xutuc [1], Marcia Cristina Teixeira Martins [4,†], María del Pilar Díaz [2,5], Gina Segovia-Siapco [6,*,†], Sandaly Oliveira da Silva Pacheco [1,*,†] and Fabio Juliano Pacheco [1,*,†]

[1] Interdisciplinary Center for Research in Health and Behavioral Sciences, School of Medicine and Health Sciences, Universidad Adventista del Plata, Libertador San Martín 3103, Argentina; sara.leeson@uap.edu.ar (S.L.); belen.carlino@uap.edu.ar (B.C.); ismael.contreras@uap.edu.ar (I.A.C.-G.); daniel.xutuc@uap.edu.ar (D.X.)

[2] School of Nutrition, Faculty of Medical Sciences, Universidad Nacional de Córdoba, Córdoba 5016, Argentina; pdiaz@fcm.unc.edu.ar

[3] School of Agricultural Sciences, Food Sciences and Engineering, Universidad Nacional de Entre Ríos, Oro Verde 3100, Argentina

[4] Graduate Department, Universidad Adventista de Chile, Camino a Las Mariposas, Chillán 11771, Chile; marciactm@yahoo.com.br

[5] Institute of Health Sciences Research (INICSA), School of Medical Sciences, Universidad Nacional de Córdoba, Córdoba 5016, Argentina

[6] School of Public Health, Loma Linda University, 24951 Circle Dr. Nichol Hall, Loma Linda, CA 92350-1718, USA

* Correspondence: rocio.gili@uap.edu.ar (R.V.G.); gsiapco@llu.edu (G.S.-S.); sandaly.oliveira@uap.edu.ar (S.O.d.S.P.); fabio.pacheco@uap.edu.ar (F.J.P.)

† These authors contributed equally to this work.

Supplementary Materials - Figure S1: eFFQ question example.

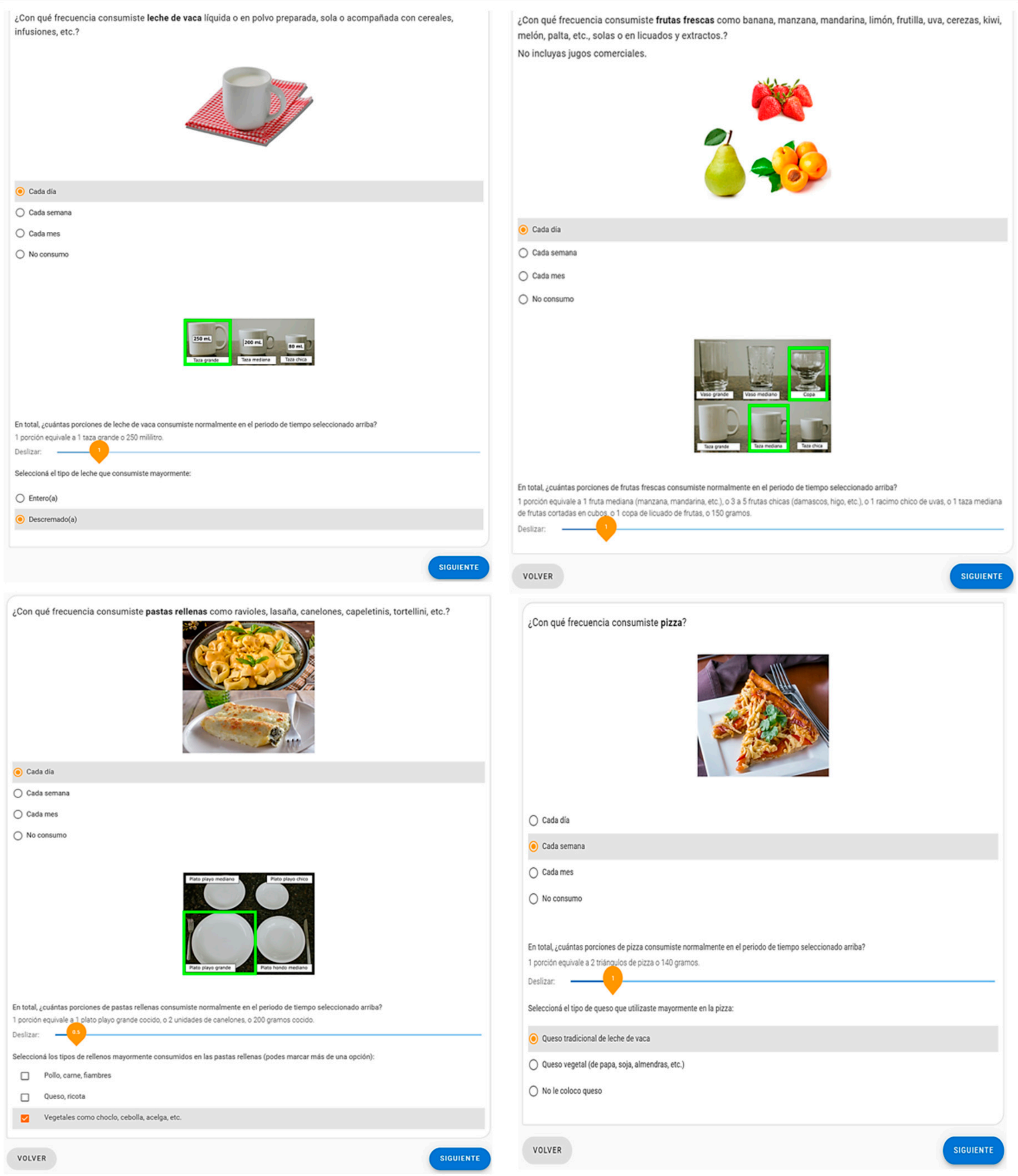

Supplement: Supplementary file 1 [file nutrients-16-01564-s001.zip › nutrients-2992475-supplementary.pdf]
